# Theoretical investigation on the linear and nonlinear optical properties of DAPSH crystal

**DOI:** 10.1038/s41598-023-35442-8

**Published:** 2023-05-27

**Authors:** Clodoaldo Valverde, Renato Medeiros, Leandro R. Franco, Francisco A. P. Osório, Marcos A. Castro, Tertius L. Fonseca

**Affiliations:** 1 Campus de Ciências Exatas e Tecnológicas, Universidade Estadual de Goiás, Anápolis, GO 75001-970 Brazil; 2 Universidade Paulista, Goiânia, GO 74845-090 Brazil; 3 Department of Engineering and Physics, Karlstad University, 65188 Karlstad, Sweden; 4 Instituto de Física, Universidade Federal de Goiás, Goiânia, GO 74690-900 Brazil; 5 Pontifícia Universidade Católica de Goiás, Goiânia, GO 74605-010 Brazil

**Keywords:** Optics and photonics, Physics

## Abstract

The linear polarizability, first and second hyperpolarizabilities of the asymmetric unit of DAPSH crystal are studied and compared with available experimental results. The polarization effects are included using an iterative polarization procedure, which ensures the convergence of the dipole moment of DAPSH embedded within a polarization field generated by the surrounding asymmetric units whose atomic sites are considered as point charges. We estimate macroscopic susceptibilities from the results of the polarized asymmetric units in the unit cell, considering the significant contribution of the electrostatic interactions in crystal packing. The results show that the influence of the polarization effects leads to a marked decrease of the first hyperpolarizability, compared with the respective isolated counterpart, which improves the concordance with the experiment. There is a minor influence of polarization effects on the second hyperpolarizability but our estimated result for the third-order susceptibility, related to the NLO process of the intensity dependent refractive index, is significant as compared with the results for other organic crystals, such as chalcone-derivatives. In addition, supermolecule calculations are conducted for explicit dimers in presence of the electrostatic embedding to illustrate the role played by the electrostatic interactions in the hyperpolarizabilities of the DAPSH crystal.

## Introduction

Molecular crystals that exhibit high second order nonlinear optical susceptibility (${\chi }^{2})$ are of great interest for applications in optical devices, and recently have been considered as efficient terahertz (THz) emitters due to the optical rectification process. Such materials can only be achieved if the chromophores are non-centrosymmetrically oriented. This arrangement is a significant challenge in synthesizing these materials, as majority of the organic compounds crystallize in centrosymmetric space groups. A range of organic stilbazolium-based crystals has been developed with the prospect that they might afford large second-order nonlinearities, since DAST (4-N,N-dimethylamino-4′-N′-methyl-stilbazolium tosylate) crystal was first reported in 1989^1,2^. DAST, an ionic organic crystal, is widely recognized as a highly favorable material for nonlinear optical (NLO) applications, particularly in electro-optics and the generation of broadband THz waves^3–6^. The crystal structure features a stilbazolium-type cation that is characterized by a donor–π–acceptor (D–π–A) architecture with intramolecular charge transfer (ICT). In such crystals, the strong Coulomb interactions that exist among the ionic species can lead to non-centrosymmetric molecular ordering, which need to be considered for an appropriated description of the NLO output of an organic salt. However, Cole et al.^7^ have shown that the presence of cation···anion interactions in DAST could affect the overall first hyperpolarizability, *β*. Their results showed that the C–H···O and C–H···C(π) cation···anion interactions have a negative impact on *β*. They proposed that the second-order nonlinear optical performance of DAST derivatives could be systematically improved by removing cation–anion interactions. A DAST-derivative of particular interest, the ionic organic DAPSH (4-N,N-dimethylamino-4′-N′-phenyl-stilbazolium hexafluorophosphate) crystal was found to be very interesting for NLO applications^8–10^, and also presents experimental second order nonlinear coefficients higher than those of DAST at 1907 nm^10^. In DAPSH, the influence of the hexafluorophosphate anion is less prominent because it has zero dipole moment and small polarizability weakly interact with the cation, in contrast to the tosylate anion in DAST.

Because bulk properties of ionic crystals cannot be obtained simply by adding the properties of isolated molecules, the design of more efficient NLO materials requires models which account for the details of the structural and electronic properties of crystalline medium. Seidler et al.^11^ have reported the linear and second-order nonlinear optical susceptibilities of three ionic organic crystals, DAST, DSTMS (4-N,N-dimethylamino-4′-N′-methyl-stilbazolium 2,4,6-trimethylbenzenesulfonate), and DAPSH, calculated by adopting a two-step multi-scale procedure. Their results showed in agreement with experiment that the second-order susceptibilities of DAPSH were shown to be superior to those of DAST and DSTMS. Ashcroft et al.^12^ have used a multipolar structural analysis of x-ray diffraction data on ionic crystals to model these organic salts within their crystalline lattice environment. From crystal structure information, structure–property relationships about DAST-based compounds have been presented for the design of new DAST derivatives with better tailored second-order NLO properties. For DAPSH crystal, Kim et al.^13^ have shown that the relative distribution of the hexafluorophosphate anions in the vicinity of the cation can affect the hyperpolarizability of the stilbazolium-type cation.

There is a large panel of methods for determining electric properties of molecules under the influence of condensed environments, based on supermolecule approaches^14,15^ or electrostatic interaction schemes^16–19^. In 2010^20^, we have proposed an interactive model for polarizing molecules in crystalline environments. This model relies on the convergence of the molecular dipole moment, enabling it to accurately calculate the (hyper)polarizabilities of molecules in crystalline phase. The initial compound investigated was L-arginine phosphate monohydrate, and the same approach was also used in the analysis of linear and nonlinear susceptibilities of urea and thiourea crystals. This iterative polarization approach which consists in describing the inhomogeneous polarizing field of the surrounding molecules, treated by point charges that represent their charge distributions, has been applied for estimating the linear and nonlinear susceptibilities of a series of new nonionic organic crystals^21–25^, and more recently, the third-order nonlinear susceptibility of ionic organic crystal, VSNS (stilbazolium derivative, 2-[2-(3-hydroxy-4-methoxy-phenyl)-vinyl]-1-methyl-pyridinium naphthalene-2-sulfonate dihydrate^26^.

In the present study, we have evaluated the in-crystal dipole moment, linear polarizability and first and second hyperpolarizabilities of the asymmetric unit of DAPSH crystal by employing the iterative polarization scheme^20^. This polarization procedure is based on the fact that the dominant intermolecular interactions are electrostatic in nature and it considers the long-range electrostatic effects, especially suitable for ionic crystals. It is worth noting that this approach does not include nonadditive intermolecular effects when the molecules are packed to form the crystals due to its huge computational cost. In this scheme, macroscopic susceptibilities have been estimated considering that the molecular properties of the polarized asymmetric units in the unit cell are assumed to be additive. Theoretical calculations for DAPSH crystal are compared to the available experimental results. In addition, by using dimer models selected from Hirshfeld surface, we investigate how specific intermolecular interactions affect the electric properties of the crystal in the presence of embedding charges. This compound is a technologically important material with possible applications in THz generation by optical rectification and related sectors^9,10^.

## Results and discussion

### In-crystal dipole moment

**Figure 1:**
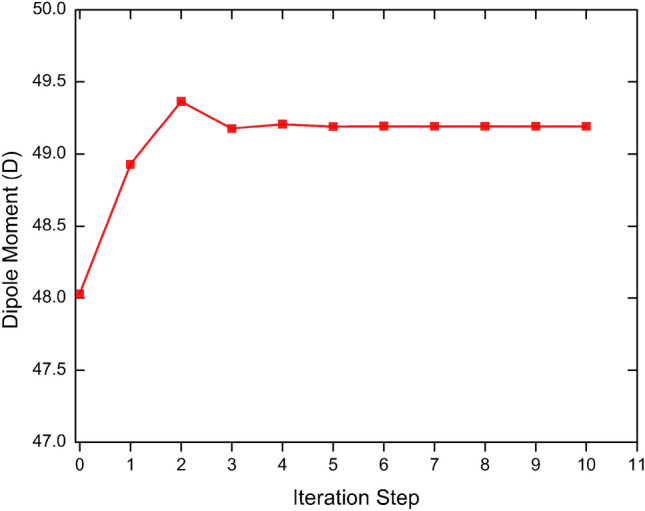
Evolution of the calculated values of the dipole moment of the asymmetric unit of DAPSH crystal with respect to the number of iterations.

The dipole moment, μ, of a single molecule embedded in a crystalline environment is not easy to be evaluated experimentally. Indirect results can be inferred in dispersion liquid with polarized electroabsorption spectra of microcrystals by using electric-field modulation spectroscopy^27^, but only scarce estimates are available. Therefore, a reliable theoretical procedure to predict dipole moments in solid phase is of great interest, and computer simulation is a natural way to carry out this type of calculation. Figure 1 shows the convergence of the total dipole moment of the asymmetric unit of DAPSH as function of the iterative step. The in-crystal μ, with CAM-B3LYP/6-311++G(d,p), converges to the value of 49.2D, 2.5% larger than the isolated value of 48.0 D. For comparison, our estimated result for μ of DAPSH is in good agreement with the B3LYP/6-31+G(d) result of the ref.^13^. It is important to highlight that the dipole moment of the DAPSH molecule is 60% larger than that of the DAST molecule, which is estimated to be 30 D^28^. In addition, the results also show that there is an intermolecular charge transfer from the N-phenyl-stilbazolium cation to the hexafluorophosphate anion, induced by the electrostatic embedding, along the polarization procedure. Our CAM-B3LYP results give for embedded [isolated] hexafluorophosphate anion a partial charge of − 1.0170e [− 1.0158], whereas for the stilbazolium-type cation a partial charge of 1.0170e [0.9901]. This is in line with the state of ionization of the hexafluorophosphate and the protonation of N-phenyl-stilbazolium in the crystalline structure, which shows some degree of ground-state charge separation^8^. Comparing with the isolated situation we note that the embedded electronic partial charge on the N-phenyl-stilbazolium cation is increased by 0.027e.

### Linear polarizability and refractive index

**Figure 2:**
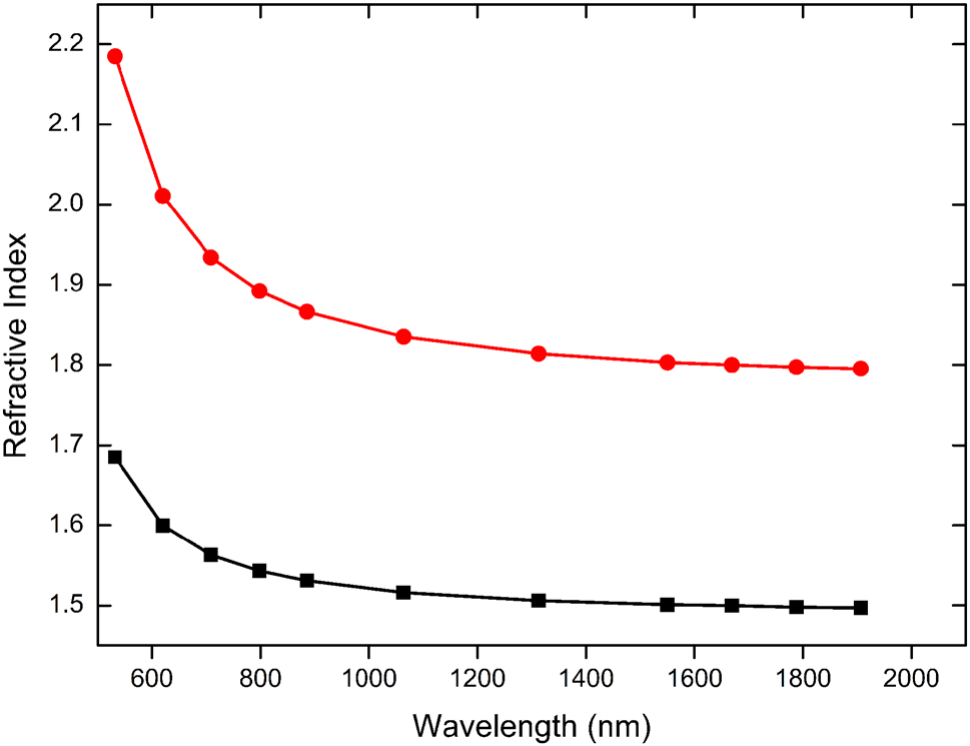
Refractive index of DAPSH crystal in the wavelength range from 532 to 1907 nm.

Table 1 shows the static and dynamic values for the average linear polarizability, $\langle \alpha \left(\omega ;\omega \right)\rangle ,$ and for the component along the dipole moment direction, ${\alpha }_{zz}\left(\omega ;\omega \right),$ of isolated and embedded DAPSH molecules, in the frequency range from $\omega =$ 0.0239 to 0.0580 a.u. ($\lambda = 1907$ to 798 nm). One can see that the environment polarization effects decrease the polarizability of the asymmetric unit. For the specified frequency interval, the decreases of $\langle \alpha \rangle$ and ${\alpha }_{zz}$ ranges from 4 to 7% and 9–13% respectively. Also shown in Table 1, the refractive index of the DAPSH crystal, which were calculated using Eq. (8), encompassing both values for the average refractive index, $\langle n\rangle$, and oriented along the dipole moment direction, ${n}_{zz}$. Figure 2 displays the dispersion curves for $\langle n\left(\uplambda \right)\rangle$ and ${n}_{zz}(\uplambda )$, which exhibit a smooth decrease of the dielectric properties with increasing wavelength. These curves are consistent with experimental data over the whole wavelength range^10^. At λ = 1907 nm, our CAM-B3LYP results for $\langle n\left(\uplambda \right)\rangle$ and ${n}_{zz}\left(\uplambda \right)$ are estimated to be ~ 1.5 and ~ 1.8, respectively. An experimental result for refractive index of DAPSH along the parallel direction to the charge-transfer axis is estimated to be ~ 2.2, at λ = 1907 nm^10^. Comparing with experiment, the CAM-B3LYP/6-311++G(d,p) model predicts for ${n}_{zz}$ a result underestimated in 18%.

**Table 1:** CAM-B3LYP/6-311++G(d,p) results for static and dynamic linear polarizability $(\mathrm{in }{10}^{-24}\mathrm{esu})$ of the asymmetric unit and refractive indexes of DAPSH crystal.

λ(nm)	$\langle \alpha \rangle$ Isolated	$\langle \alpha \rangle$ Embedded	${\alpha }_{zz}$ Isolated	${\alpha }_{zz}$ Embedded	$\langle n\rangle$ Embedded	${n}_{zz}$ Embedded
$\infty$	55.59	53.07	103.82	94.16	1.490	1.779
1907	56.83	54.05	107.12	96.65	1.497	1.795
1550	57.52	54.59	108.95	98.02	1.501	1.803
1313	58.36	55.24	111.20	99.69	1.506	1.814
1064	60.07	56.56	115.78	103.04	1.516	1.835
798	64.87	60.12	128.73	112.20	1.543	1.892

### First and second hyperpolarizabilities and susceptibilities

Static and dynamic results for the total first hyperpolarizability, ${\beta }_{tot}(-2\omega ;\omega ,\omega )$, and the dipole orientation component, ${\beta }_{zzz}(-2\omega ;\omega ,\omega )$, of the isolated and embedded molecules of DAPSH are quoted in Table 2, in the frequency range from $\omega =$ 0.0239 to 0.0428 a.u. ($\lambda = 1907$ to 1064 nm). An overall look on the results, indicates that the polarizing field effect significantly decreases the first hyperpolarizability, making its impact greater than that on the linear polarizability. This effect becomes increasingly pronounced as the wavelength decreases. Thus, at λ = 1907 nm, the dynamic value for ${\beta }_{tot}$ (${\beta }_{zzz}$) of the embedded molecule is smaller than that of isolated one by a factor of 11% (15%). Based on Stark spectroscopy data, an experimental result for the static first hyperpolarizability of DAPSH has been estimated to $135\times {10}^{-30}\mathrm{esu}$ ^8^. Compared with experiment, the static results for ${\beta }_{zzz}$ of the embedded asymmetric unit of $162.77\times {10}^{-30}\mathrm{ esu}$, is overestimated in 21%. Note that a better agreement with experiment would not have been met without accounting for the polarizing field effect, which decreases the hyperpolarizability of the cation. However, the results in Table 2 show that, for the second-order susceptibility, the polarization effects do not improve comparison with the experiment. Our in-crystal estimate for ${\chi }_{zzz}^{(2)}(-2\omega ;\omega ,\omega )=$ 79.78 pm/V, for instance, is underestimated in 87%, as compared with an experimental result for ${\chi }^{2}(-2\omega ;\omega ,\omega )$ of DAPSH estimated to be 580 ± 80 pm/V, at $\omega =$ 0.0239 a.u. (λ = 1907 nm)^10^. Although our iterative procedure provides results consistent with the solid-state phase, it does not include nonadditive intermolecular effects when the asymmetric units are packed to form the crystals and molecular hyperpolarizabilities of the embedded asymmetric units in the unit cell are assumed to be additive.

**Table 2:** CAM-B3LYP/6-311++G(d,p) results for static and dynamic first hyperpolarizability (${\mathrm{in }10}^{-30}\mathrm{esu})$ of the asymmetric unit and second-order susceptibility (in pm/V) of DAPSH crystal.

λ(nm)	${\beta }_{tot}$ Isolated	${\beta }_{tot}$ Embedded	$\left|{\beta }_{zzz}\right|$ Isolated	$\left|{\beta }_{zzz}\right|$ Embedded
$\infty$	202.44	186.09	185.31	162.77
1907	277.66	246.96	252.99	214.97
1550	335.35	291.40	304.85	253.03
1313	431.11	361.32	390.85	312.83
1064	800.41	594.18	722.17	511.67

	${\chi }_{tot}^{(2)}$ Isolated	${\chi }_{tot}^{(2)}$ Embedded	${\chi }_{zzz}^{(2)}$ Isolated	${\chi }_{zzz}^{(2)}$ Embedded
$\infty$	86.31	79.34	79.00	69.40
1907	118.38	105.29	107.88	79.78
1550	142.98	124.24	129.97	107.88
1313	183.80	154.05	166.64	133.38
1064	341.26	253.33	307.90	218.15

**Figure 3:**
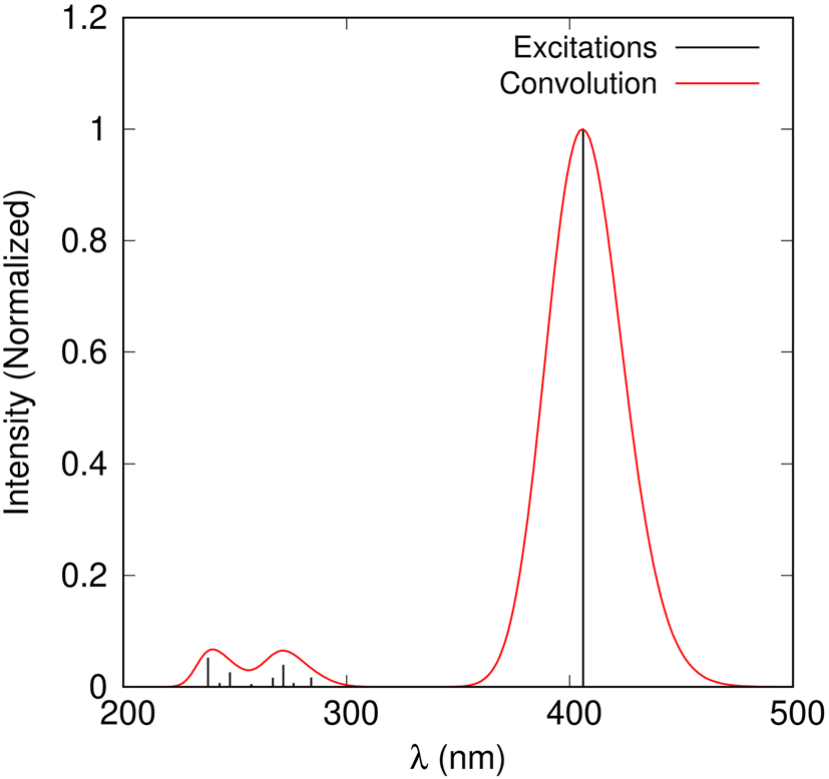
Theoretical UV–Vis absorption spectrum of the asymmetric unit of DAPSH crystal. Gaussian convolution with full width at half maximum (FWHM) of 0.3 eV.

**Figure 4:**
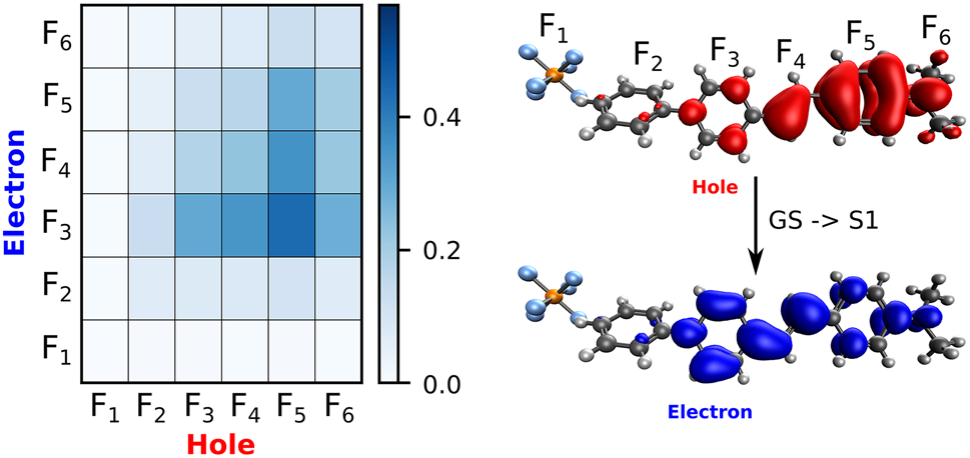
Transition density matrix (TDM) of the most intense electronic excitation (from ground state to the first excited state) of the asymmetric unit of DAPSH crystal.

In Fig. 3, we present the absorption spectrum of the embedded asymmetric unit. The TD-CAM-B3LYP/6-311++G(d,p) calculations show the presence of one dominant transition, indicating that the optical properties are characterized by π–π^*^ transition involving a HOMO → LUMO excitation. Thus, we have analyzed the features of the first excited state of DAPSH, which is inherently connected to the first hyperpolarizability, in terms of the localization of the excitation hole and excited electron or the presence of charge transfer^29,30^. This analysis was conducted using fragment-based techniques implemented in the TheoDORE package^31^. We have partitioned into 6 fragments to obtain the charge transfer numbers of asymmetric unit, as shown in Fig. 4: the dimethylamino (DMA) group (6), the rings A (5), B (3) and C (2), bridge (4) and the hexafluorophosphate (1). Two-dimensional matrix (Ω) plots of the charge transfer numbers describing the interaction of molecular fragments are also displayed in Fig. 4. One can see that the shape of the frontier orbitals is delocalized over the fragments 3, 4, 5 and 6. The excited state is characterized by two contributions located on the diagonal, corresponding to local excitations on the fragments 5 and 3, and by three off-diagonal contributions, with the other fragments playing a secondary role. There is a significant charge transfer to fragments 3 and 4, with a dominant excitation hole located on fragment 5. A similar conclusion has been drawn for the changes in the electronic density distribution of the dominant excited state of phenol blue (a typical merocyanine dye) in solution^32^.

In Table 3, we report the static and dynamic results for the average second hyperpolarizability, $\langle \gamma \left(-\omega ;\omega ,\omega ,\omega \right)\rangle$, (related to the third-order NLO processes of IDRI) of the isolated and embedded DAPSH molecule. Unlike what was observed for the first hyperpolarizability, the impact of polarization effects on $\langle \gamma \left(-\omega ;\omega ,\omega ,\omega \right)\rangle$ values is mild, in the range of $\uplambda =\mathrm{\infty and }1550\mathrm{ nm}$, resulting in increases of 6%, compared to the isolated values. This effect remains small even at shorter wavelengths, where occur a reduction in the values of $\langle \gamma \left(-\omega ;\omega ,\omega ,\omega \right)\rangle$. The influence of dispersion on the hyperpolarizability is notable, and its impact is relatively reduced in the crystalline medium. From $\lambda$ = 1907 to 1064 nm, the CAM-B3LYP results for $\langle \gamma \left(-\omega ;\omega ,\omega ,\omega \right)\rangle$ of embedded DAPSH molecule are augmented, with respect to static ones, by factors of 24% and 94%. For comparison, our prediction for $\langle \gamma \left(-\omega ;\omega ,\omega ,-\omega \right)\rangle$ , at $\uplambda = 1907\mathrm{nm},$ is 62% larger than the value of the ionic organic crystal (VSNS) which is estimated to be $258\times {10}^{-36}\mathrm{esu}$ ^26^.

**Table 3:** Static and dynamic results for the second hyperpolarizability (in ${10}^{-36}\mathrm{esu})$ of the asymmetric unit and third order nonlinear susceptibility (in ${10}^{-20}{\mathrm{m}}^{2}/{\mathrm{V}}^{2})$ of DAPSH crystal at the CAM-B3LYP/6-311++G(d,p) level.

λ(nm)	$\langle \gamma \rangle$ Isolated	$\langle \gamma \rangle$ Embedded	$\langle {\chi }^{(3)}\rangle$ Isolated	$\langle {\chi }^{\left(3\right)}\rangle$ Embedded
$\infty$	320.49	338.10	0.91	0.96
1907	408.44	418.28	1.16	1.19
1550	461.00	465.31	1.31	1.32
1313	529.51	525.71	1.51	1.50
1064	682.66	657.41	1.94	1.87

It is instructive to compare our theoretical results for the third-order nonlinear susceptibility with available theoretical and experimental results of other organic crystals, that may have potential for application in NLO processes. The CAM-B3LYP/6-311++G(d,p) results for the third-order nonlinear susceptibility, $\langle {\chi }^{\left(3\right)}\left(-\omega ;\omega ,\omega ,-\omega \right)\rangle$, of DAPSH crystal calculated from the hyperpolarizabilities of the polarized asymmetric units in the unit cell, are also quoted in Table 3. For comparison, we have considered theoretical results that were obtained using the same iterative scheme. Our estimated values for ${\chi }^{\left(3\right)}\left(-\omega ;\omega ,\omega ,-\omega \right)$ are significant compared to the results obtained for other organic crystals, such as chalcone-derivatives, which generally fall within the range of 10^−22^–10^−20^ m^2^/V^2^^21–25^. In particular, the experimental result for ${\chi }^{\left(3\right)}\left(-\omega ;\omega ,\omega ,-\omega \right)$ of (2E)-3-(3-methylphenyl)-1-(4-nitrophenyl)prop-2-en-1-one (3MPNP), a crystal used as a reference, is estimated to be $2.77\times {10}^{-20} {\mathrm{m}}^{2}/{\mathrm{V}}^{2}$, at $\uplambda = 532\mathrm{ nm}$ ^33^.

**Figure 5:**
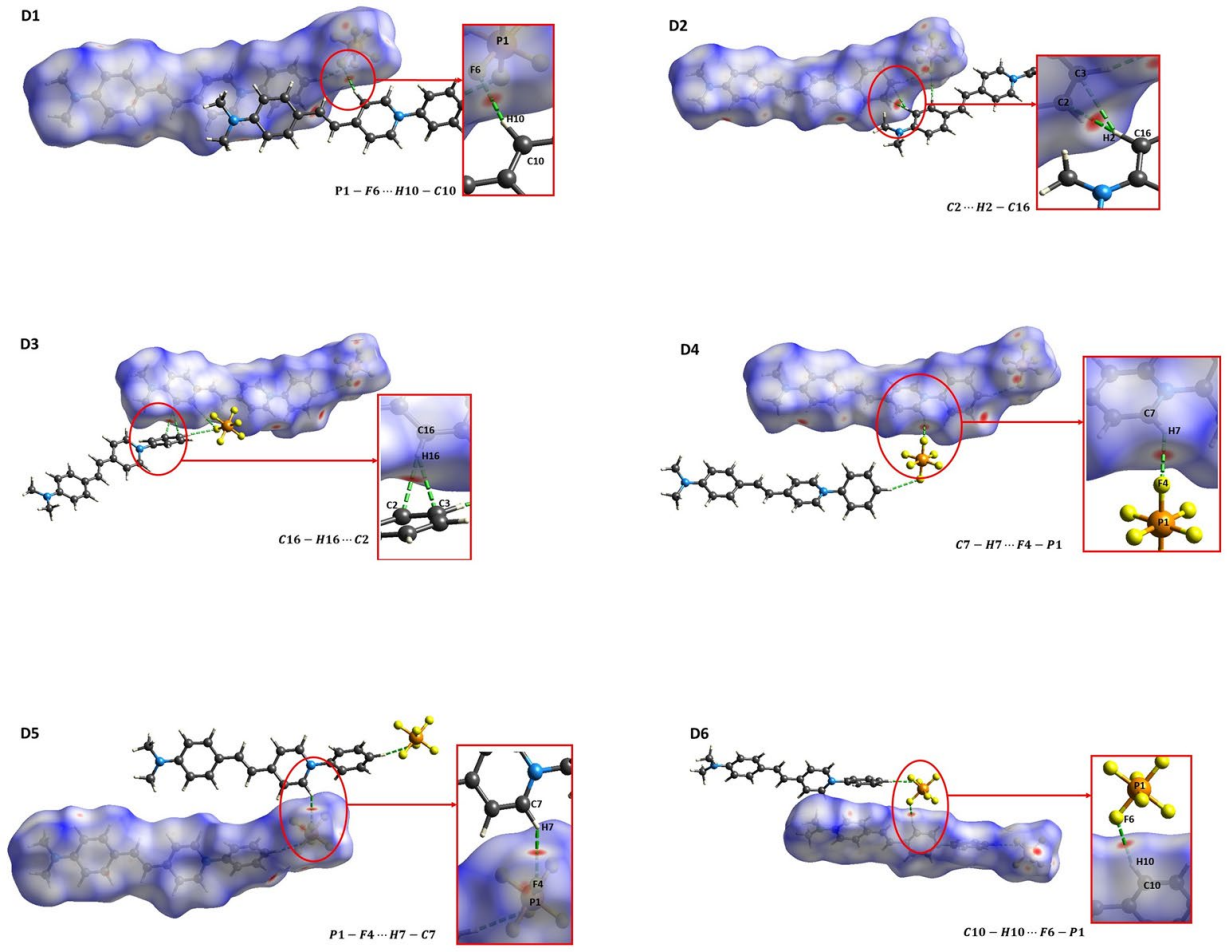
Embedded asymmetric unit dimers selected from Hirshfeld surface analysis.

In addition, by using the supermolecule approach and iterative polarization procedure, we investigate how the effect of the intermolecular interactions modify the first and second hyperpolarizabilities of DAPSH in presence of embedding charges. Hirshfeld surface^34,35^ analysis was employed to explore the noncovalent interactions that are responsible for crystal packing and to select the embedded asymmetric unit dimers (see Fig. 5). These supermolecule calculations partially account for the exchange and dispersion effects. We present CAM-B3LYP/6-311++G(d,p) results for the nonlinear properties of embedded dimers in Table 4. One can see that intermolecular interactions can either enhance or reduce of hyperpolarizabilities upon crystallization. In particular, C_10_–H_10_···F_6_–P_1_ (D1) and C_7_–H_7_···F_4_–P_1_ (D4) interactions between cations and anions in DAPSH crystal have a beneficial effect for ${\beta }_{zzz}$. For the static (dynamic) results of ${\beta }_{zzz}$, the increases due to these interactions are of 20% and 13% (22% and 14%), relative to the single asymmetric unit, which is the embedded monomer. In addition, such interactions result in increases of 29% and 23% and (31% and 24%) for $\langle \gamma \rangle$. These observed increases are in line with the findings of Kim et al.^13^ regarding the intrinsic first hyperpolarizability of cation chromophores. They found that extrinsic isotropic point-charges or anions have almost no effect on this property, and any resulting increase in magnitude is limited to approximately 15%. However, the effect of the C_7_–H_7_···F_4_–P_1_ (D5) interaction has a minor impact, leading to small reductions of 4% and 7% on the values of ${\beta }_{zzz}$ and $\langle \gamma \rangle$, respectively. Differently, the frequency-dependent results of ${\beta }_{tot}$ show that all considered intermolecular interactions lead to increases that range from 15 to 48%.

**Table 4:** Static and dynamic results for the first hyperpolarizability (${\mathrm{in }10}^{-30}\mathrm{esu})$ and second hyperpolarizability (${\mathrm{in }10}^{-36}\mathrm{esu})$ of embedded DAPSH dimers, D1, D2, D3, D4, D5 and D6, selected from Hirshfeld surface at the CAM-B3LYP/6-311++G(d,p) level.

$\uplambda (\mathrm{nm})$	${\beta }_{tot}$	${\beta }_{tot}$/2					
	Monomer	D1	D2	D3	D4	D5	D6
∞	186.08	222.67	204.56	176.84	205.44	176.25	224.78
1907	203.49	301.00	274.33	234.09	275.64	233.25	304.18

	$\left|{\beta }_{zzz}\right|$	$\left|{\beta }_{zzz}\right|$/2					
∞	162.77	194.41	183.42	155.89	184.12	155.45	196.38
1907	214.97	261.52	245.17	205.38	246.22	204.72	264.45

	$\langle \gamma \rangle$	$\langle \gamma \rangle$/2					
∞	338.10	435.82	416.85	313.80	416.98	313.84	436.20
1907	418.28	548.64	519.68	387.33	520.11	387.24	549.80

In summary, the dipole moment of the asymmetric unit of DAPSH crystal was determined by using an iterative electrostatic polarization scheme, that has been previously reported^20,36^, in which the surrounding asymmetric units are represented by point charges. Our study provides a first estimation of the macroscopic susceptibilities, using results from the polarized asymmetric units located in the unit cell. This estimation takes into consideration the substantial influence of electrostatic interactions on the arrangement of crystal. It is found that the result for the average dipole moment of asymmetric unit, with CAM-B3LYP/6-311++G(d,p), converges to the value of 49.2 D. This is 2.5% larger than the isolated value of 48.0 D. The impact of polarization effects on the first hyperpolarizability is marked and leads to improved concordance with the experiment, with a result for |${\beta }_{zzz}$| of embedded asymmetric unit that exceed the measured result by only 21%. However, the calculated second-order nonlinear susceptibility is significantly underestimated, as compared with the experiment. For the second hyperpolarizability, the polarization effects have a negligible impact but the estimated result for the third-order susceptibility, related to the NLO process of the intensity dependent refractive index, is significant as compared with the results for other organic crystals, such as chalcone-derivatives. In addition, supermolecule calculations, which partially consider exchange and dispersion effects, were conducted for explicit dimers in presence of the electrostatic embedding to analyze the influence of intermolecular interactions on the in-crystal NLO properties. It turns out that intermolecular interactions between cations and anions can have a significant effect on the static and dynamic (at λ = 1907 nm) values of the hyperpolarizabilities of the DAPSH crystal but a further investigation using different sizes of embedded clusters is necessary for a more comprehensive analysis.

## Methods

**Figure 6:**
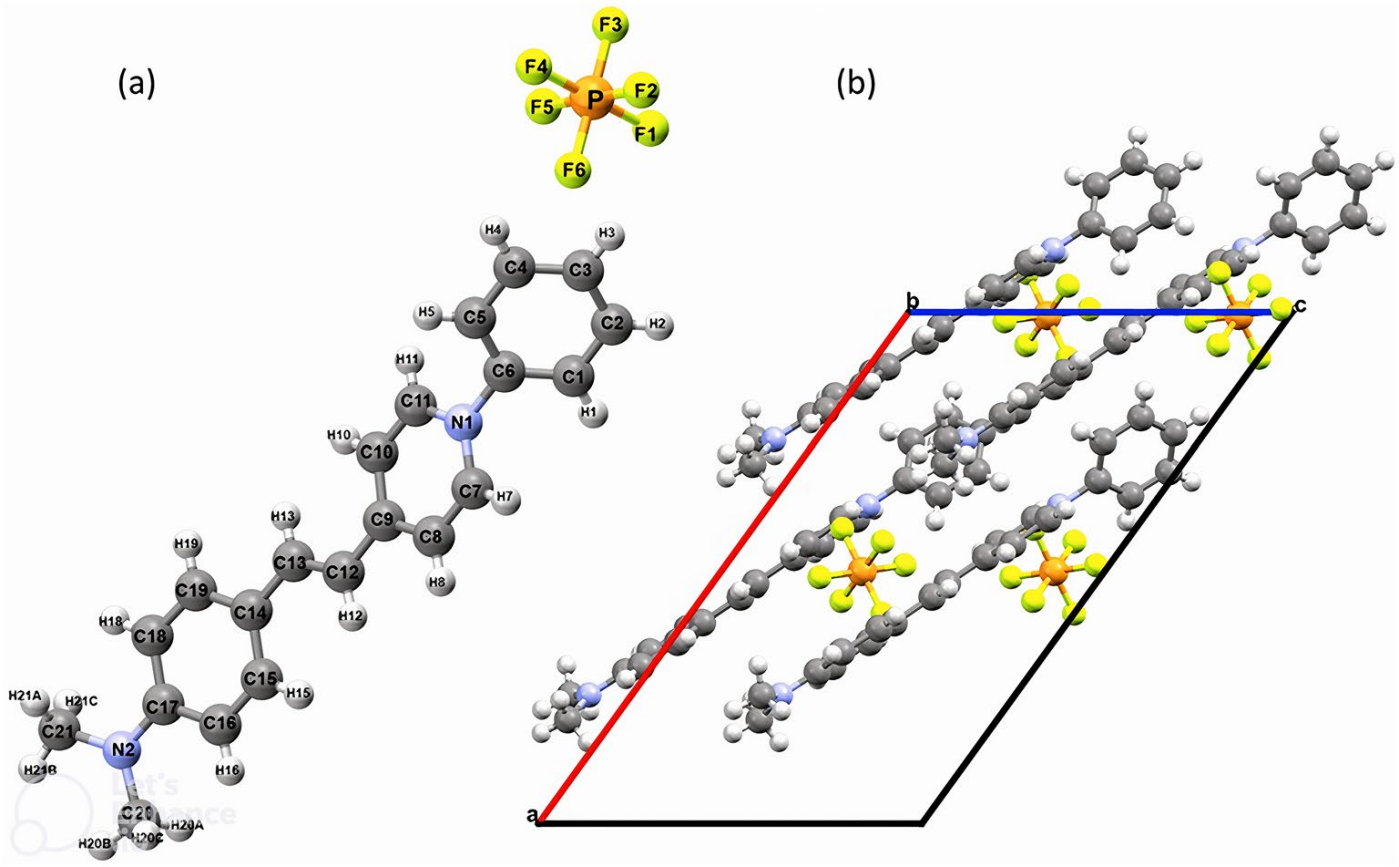
(**a**) Asymmetric unit of DAPSH with the numbering used in this study. (**b**) Projection along the *b* axis of the unit cell of DAPSH.

The DAPSH crystal belongs to the monoclinic crystalline system and non-centrosymmetric C*c* space group^10^. Figure 6 shows the asymmetric unit of DAPSH and the respective unit cell with four asymmetric units. The lattice parameters and the angles are *a* = 19.384Å, b=10.636Å, *c* = 11.784Å, and $\alpha =\gamma =90^\circ \mathrm{and} \beta =125.93^\circ$, respectively. The unit cell volume is V =1967.24 Å^3^ and the molecular formula is ${\mathrm{H}}_{21}{\mathrm{C}}_{21}{\mathrm{F}}_{6}{\mathrm{N}}_{2}\mathrm{P}$. The presence of hexafluorophosphate counterions has been found to favor the formation of alternating cationic and anionic sheets, with parallel alignment of N-phenyl-stilbazolium chromophores. Within a polar bulk structure, the anions are located close to the electron-deficient pyridinium rings. The DAPSH geometry was obtained from a crystallographic information file (CIF) and no further geometry optimization was performed in the subsequent quantum mechanical calculations. The DAPSH structure was deposited at Cambridge Crystallography Data Centre (CCDC) with the code 163,560.

### Asymmetric unit polarization

We have developed a procedure to describe the electronic polarization of an asymmetric unit^20^ or unit cell^36^ in organic crystals. In this procedure we have iteratively applied the electrostatic embedding approach to calculate the dipole moment of the asymmetric unit of DAPSH, immersed in a bulk with dimensions of 13 × 13 × 13 unit cells with four asymmetric units each, totalizing with 448,188 atoms. It should be stressed that no periodic boundary conditions were applied for evaluating electronic properties in the solid phase. However, test calculations have shown that a cluster of at least 9 × 9 × 9 unit cells is large enough to assure the convergence of the in-crystal dipole moment. The iterative procedure is started with partial atomic charges that were obtained by using an electrostatic potential fit (CHELPG^37^, as implemented in the Gaussian 16 package^38^) from the CAM-B3LYP/6-311++G(d,p) charge density of the isolated asymmetric unit. These charges were placed on all the corresponding atomic sites of the molecules surrounding the explicit asymmetric unit. Another calculation is then performed in the presence of the electrostatic embedding to obtain the new values of the atomic charges. This procedure is iterated until the convergence of the in-crystal dipole moment is obtained. A similar iterative approach has been successfully applied in the study of the polarization of organic molecules in aqueous solution^39–43^.

### Hirshfeld surface

The Hirshfeld surface (HS) is a spatial map that allows visualize the space occupied by a molecule in the crystal as determined by its electron density^34,35^. HS is a valuable tool for quantifying the proximity of atoms or molecules in a crystal. It achieves this by providing a surface that is equidistant from two neighboring atoms, allowing the distance between them to be estimated by analyzing the shape and size of the surface. This feature is particularly useful for investigating intermolecular interactions such as hydrogen bonding, van der Waals contacts, and $\pi -\pi$ stacking interactions, among others. Through of a color mapping the short (red) and longer (blue) contacts can be visualized. HS was plotted via CrystalExplorer17^44^ to provide information about the intermolecular interactions in DAPSH crystal.

### Electro-optical parameters

The total dipole moment, average linear polarizability and the total first hyperpolarizability related to the second-harmonic generation, were calculated through the expressions,

1 $$\mu ={\left({\mu }_{x}^{2}+{\mu }_{y}^{2}+{\mu }_{z}^{2}\right)}^\frac{1}{2},$$

2 $$\left\langle \alpha (-\omega ;\omega )\right\rangle =\frac{1}{3}\sum_{i=x,y,z}{\alpha }_{ii},$$

3 $${\beta }_{tot}\left(-2\omega ;\omega ,\omega \right)=\sqrt{{\beta }_{x}^{2}+{\beta }_{y}^{2}+{\beta }_{z}^{2}}$$

where

4 $${\beta }_{i}=\frac{1}{3}\sum_{j}\left({\beta }_{ijj}+{\beta }_{jji}+{\beta }_{jij}\right) \quad i,j=(x,y,z)$$

The average second hyperpolarizability corresponding to the dc-Kerr effect was calculated using the expression,

5 $$\begin{aligned} \langle \gamma \left( { - \omega ;\omega ,{\text{0}},{\text{0}}} \right)\rangle = & \frac{1}{5}\left( {\gamma _{{xxxx}} + \gamma _{{yyyy}} + \gamma _{{zzzz}} } \right) \\ & \quad + \frac{1}{{15}}\left[ {\gamma _{{xxyy}} + \gamma _{{yyxx}} + \gamma _{{xxzz}} + \gamma _{{zzxx}} + \gamma _{{yyzz}} + \gamma _{{zzyy}} } \right. \\ & \quad \left. { + 4\left( {\gamma _{{yxyx}} + \gamma _{{zxzx}} + \gamma _{{zyzy}} } \right)} \right] \\ \end{aligned}$$

whereas the average second hyperpolarizability associated to nonlinear optical process of the intensity dependent refractive index (IDRI) ($\langle \gamma \left(-\omega ;\omega ,\omega ,-\omega \right)\rangle )$ for small frequencies^33,45,46^ was calculated by the expression,

6 $$\langle \gamma \left(-\omega ;\omega ,\omega ,-\omega \right)\rangle \cong 2\langle \gamma \left(-\omega ;\omega ,\mathrm{0,0}\right)\rangle -\langle \gamma \left(0;\mathrm{0,0},0\right)\rangle .$$

Static and dynamic electric properties were calculated analytically using the Hartree–Fock Perturbed Coupled (CPHF) and time-dependent Hartree–Fock (TDHF) methods with the CAM-B3LYP method using the 6-311++G(d,p) basis set, as implemented in the Gaussian 16 package^38^.

We have estimated the linear susceptibility and the refractive index as well as the second and third-order susceptibilities by using the following relations:

7 $$\langle {\chi }^{(1)}\left(-\omega ;\omega \right)\rangle =\frac{N\langle \alpha \left(-\omega ;\omega \right)\rangle }{{\varepsilon }_{o}V},$$

8 $${n}_{ij}\left(\omega \right)=\sqrt{1+\langle {\chi }^{\left(1\right)}\left(-\omega ;\omega \right)\rangle },$$

9 $${\chi }^{(2)}\left(-2\omega ;\omega ,\omega \right)=\frac{N\beta \left(-2\omega ;\omega ,\omega \right)}{{\varepsilon }_{o}V},$$

and

10 $$\left\langle {\chi }^{\left(3\right)}\left(-\omega ;\omega ,\omega ,-\omega \right)\right\rangle = \frac{N \langle \upgamma \left(-\omega ;\omega ,\omega ,-\omega \right)\rangle }{{\epsilon }_{o}V},$$

where ${\epsilon }_{o}$ is the vacuum permittivity, *N* is the number of asymmetric units in the unit cell and *V* is the unit cell volume.

Excitation energies were obtained with time dependent density functional theory (TDDFT) at the CAM-B3LYP/6-311++G(d,p) level, as implemented in the Gaussian 16 package^38^. CAM-B3LYP was chosen because it is a hybrid exchange–correlation functional that considers long-range correction, which makes it an effective functional routinely employed in calculations of these properties^47–49^. To assess the quality of the basis set used, we performed a test calculation with 6-311++G(2d,2p) and obtained the result ${181.55 \times 10}^{-30 }\mathrm{esu}$ for |${\beta }_{zzz}$|. The difference in relation to the value obtained with 6-311++G(d,p), quoted in Table 2, is only 2%, which indicates that 6-311++G(d,p) is a suitable basis set.

## Data Availability

All data generated or analyzed during this study has been provided in the manuscript.
